# Does pain hurt more in Spanish? The neurobiology of pain among Spanish–English bilingual adults

**DOI:** 10.1093/scan/nsad074

**Published:** 2023-12-15

**Authors:** Morgan Gianola, Maria M Llabre, Elizabeth A Reynolds Losin

**Affiliations:** Psychology, University of Miami, Coral Gables, Florida 33146, USA; Psychology, University of Miami, Coral Gables, Florida 33146, USA; Biobehavioral Health, Penn State University, State College, Pennsylvania 16801, USA

**Keywords:** bilingual, pain, Hispanic/Latino, culture, fMRI

## Abstract

We previously found Spanish-English bilingual adults reported higher pain intensity when exposed to painful heat in the language of their stronger cultural orientation. Here, we elucidate brain systems involved in language-driven alterations in pain responses. During separate English- and Spanish-speaking fMRI scanning runs, 39 (21 female) bilingual adults rated painful heat intermixed between culturally evocative images and completed sentence reading tasks. Surveys of cultural identity and language use measured relative preference for US-American *vs* Hispanic culture (cultural orientation). Participants produced higher intensity ratings in Spanish compared to English. Group-level whole-brain differences in pain-evoked activity between languages emerged in somatosensory, cingulate, precuneus and cerebellar cortex. Regions of interest associated with semantic, attention and somatosensory processing showed higher average pain-evoked responses in participants’ culturally preferred language, as did expression of a multivariate pain-predictive pattern. Follow-up moderated mediation analyses showed somatosensory activity mediated language effects on pain intensity, particularly for Hispanic oriented participants. These findings relate to distinct (‘meddler’, ‘spotlight’ and ‘inducer’) hypotheses about the nature of language effects on perception and cognition. Knowledge of language influences on pain could improve efficacy of culturally sensitive treatment approaches across the diversity of Hispanic adults to mitigate documented health disparities in this population.

## Introduction

### Background

Mounting experimental evidence demonstrates that culture and language meaningfully impact behavioral and neurocognitive outcomes. For instance, ‘cultural frame switching’ studies causally link cultural mindsets to various psychological (Chattaraman *et al.*, 2010; Kreitler and Dyson, 2016) and neural processes (Chiao *et al.*, 2010; Ng *et al.*, 2010; Cheon *et al.*, 2013) by priming multicultural individuals with culturally salient images (Chattaraman *et al.*, 2010; Ng *et al.*, 2010; Kreitler and Dyson, 2016) and/or specific languages (Verkuyten and Pouliasi, 2002; Kemmelmeier and Cheng, 2004; Lechuga, 2008). Additionally, ‘linguistic relativity’ studies connect grammar and linguistic patterns to variation in perceptual and neurocognitive processes across speakers of distinct languages (e.g. Papafragou *et al.*, 2008; Boroditsky and Gaby, 2010; Boutonnet *et al.*, 2013). This framework is extended into bilingual populations, revealing within-individual cognitive shifts across languages (e.g. Schonberg *et al.*, 2019), such as altered personality report and increased helping behaviors in Spanish compared to English contexts (Ramírez-Esparza *et al.*, 2008). Thus, behavioral and neurobiological processes exhibit sensitivity to language and cultural context within multilingual/multicultural persons.

Pain, a clinically relevant perception involved in most serious medical complaints (Institute of Medicine [IOM US] Committee on Advancing Pain Research, Care, and Education, 2011), is modulated by social (e.g. Hunter *et al.*, 2014; Koban and Wager, 2016) and psychological processes such as attention (Legrain *et al.*, 2009), expectations (Czerniak *et al.*, 2016), anxiety (Tang and Gibson, 2005) and stress (Cathcart *et al.*, 2008; Mertens *et al.*, 2020). These same psychological processes display variability across cultural and/or linguistic contexts (Guttfreund, 1990; Flecken *et al.*, 2014; Ruiz *et al.*, 2018). While limited research addresses variation in pain processing across languages, explicit ratings and neural metrics of pain empathy are affected by language conditions among Chinese–English bilingual study participants (Wu *et al.*, 2020). Moreover, Swiss chronic back pain patients from distinct language groups express systematic differences in preferred pain coping strategies (Schulz *et al.*, 2013). These findings contribute to growing evidence for pain modulation across social, cultural and language contexts (see Anderson and Losin, 2016 for review).

Our investigation into language-pain associations was targeted to Hispanic^1^ Americans for several reasons. Hispanics comprise the largest bilingual group in the United States (U.S. Census Bureau, 2017), endorse higher levels of multiculturalism than non-Hispanic whites (Ryan *et al.*, 2010) and show salient priming of cultural frames by language context (Lechuga and Wiebe, 2009; Lee *et al.*, 2010). The interplay between language and pain may be particularly pertinent since this population faces disparities in healthcare access (Nguyen *et al.*, 2005), pain assessment (Meghani *et al.*, 2012) and treatment (K. Anderson *et al.*, 2009; Lee *et al.*, 2019). Language may also contribute to the ‘Hispanic Paradox’: lower all-cause mortality and equivalent health outcomes (e.g. cancer rates, mental health) among US Hispanics compared to non-Hispanic whites, despite greater risk factors, including lower socioeconomic status, educational attainment and occupational opportunity (Gallo *et al.*, 2009 and see Velasco-Mondragon *et al.*, 2016 for review). Research suggests cultural factors such as diet and social cohesion may connect to this paradox (Velasco-Mondragon *et al.*, 2016; Ruiz *et al.*, 2018). Ruiz *et al.* (2018) report evidence that culturally informed appraisals can reduce stress accumulation among Hispanics, while Spanish’s emotional lexicon is hypothesized to contribute to reduced stress reactivity (Llabre, 2021). Hence, the Hispanic population’s health resiliency likely emerges from several sociocultural factors, one of which (Spanish language) we investigate here.

### Previous findings and potential mechanisms

We previously investigated how bilingual adults’ pain responses differed across English *vs* Spanish contexts (Gianola *et al.*, 2021). Briefly, 80 bilingual participants rated painful heat during separate English and Spanish experimental sessions, both before and after language-congruent cultural priming (developed from Gianola *et al.*, 2020). Acculturation surveys measured participants’ relative orientation toward Hispanic and US-American culture. Overall, participants exhibiting asymmetric cultural orientation (i.e. greater identification with Hispanic relative to US-American culture, or vice versa) produced higher pain intensity and physiological arousal responses in the language of their stronger cultural orientation, irrespective of cultural priming. Utilizing Wolff and Holmes’s (2011) framework, we hypothesized three mechanisms for these language-pain effects, each implicating distinct neural systems: language as spotlight (attention), inducer (somatosensory/nociception) and meddler (semantic processing).

‘Language as spotlight’ suggests distinct words and constructions across languages could differentially highlight the pain experience, thus recruiting more attentional resources in participants’ preferred language. Such effects might arise through prefrontal attentional control structures including the anterior cingulate (ACC; Kuner and Kuner, 2020). This conflict monitoring region modulates its activity during pain coping (Emmert *et al.*, 2017), shows higher efficiency in bilingual adults (Abutalebi *et al.*, 2012) and engages during bilingual language selection and switching (Hernandez, 2009; van Heuven and Dijkstra, 2010; Abutalebi and Green, 2016). Similarly, structures involved in top-down attention shifting and sustained awareness (i.e. middle frontal gyrus [MFG], superior parietal lobule [SPL]), implicated in bilingual language control, also contribute to attentional pain modulation (Wiech *et al.*, 2005; Bushnell *et al.*, 2013; Japee *et al.*, 2015). Hence, if English and Spanish differentially ‘spotlight’ pain experiences, we might observe modulated pain-evoked activity in attention-directing structures across languages.

Alternatively, languages may prime distinct modes of schematic processing which remain engaged even without explicit language use (language as inducer), such as increased arousal in participants’ preferred language. In this case, higher intensity ratings may arise from greater nociceptive activity, implicating the sensory-discriminative lateral spinothalamic pathway (Kuner and Kuner, 2020), including primary (SI) and secondary (SII) somatosensory cortex, lateral thalamus (Morton *et al.*, 2016), posterior insula (Segerdahl *et al.*, 2015) and cerebellum (Moulton *et al.*, 2010). The Neurologic Pain Signature (NPS) is a multivariate brain measure which weighs activity across primary nociceptive regions; NPS expression exhibits high sensitivity and specificity for distinguishing acute pain from other aversive stimuli (Wager *et al.*, 2013; Lindquist *et al.*, 2017; Losin *et al.*, 2020; Han *et al.*, 2022). If language ‘induces’ distinct processing modes, we expect NPS expression and average blood oxygen level dependent (BOLD) responses across somatosensory regions will differ in response to pain delivered in English and Spanish contexts.

Finally, perceptually equivalent stimuli could be rated differently between languages due to spontaneous recruitment of linguistic codes in tandem with non-linguistic pain processing (language as meddler). If so, regions involved in working memory and conditioned pain modulation, like superior frontal gyrus (SFG; Boisgueheneuc *et al.*, 2006; Alagapan *et al.*, 2019; Nahman-Averbuch and Timmers, 2022) might exhibit pain-evoked activity while engaging linguistic codes which are lexically and semantically processed in left lateralized posterior inferior, middle temporal (pITG, MTG) and inferior frontal (IFG) gyri (Costafreda *et al.*, 2006; Friederici, 2011). Since these linguistic codes differ between English and Spanish, differential activations in these regions could subsequently influence evaluations involving ventromedial prefrontal cortex (Bartra *et al.*, 2013; Mariën *et al.*, 2017). Therefore, pain-evoked activity across areas engaged in linguistic processing could clarify whether language ‘meddles’ in non-linguistic processing of pain.

### The present study

This exploratory study quantified the existence, magnitude and direction of language effects on bilingual adults’ pain. Hispanic adults rated painful heat stimulations during separate English and Spanish fMRI scans. Hispanic and US-American cultural frames were primed via both language context and language-congruent cultural images (Gianola *et al.*, 2020). BOLD signal was measured during pain rating and semantic judgment tasks, completed in both languages. Semantic judgment activity was used to define a ‘semantic’ region of interest (ROI) whose pain-evoked activity was compared across conditions. Meta-analytic maps of areas preferentially activated by attention or somatosensory tasks from Neurosynth.org (Yarkoni *et al.*, 2011) defined separate ‘attention’ and ‘somatosensory’ ROIs. Mean pain-evoked BOLD responses in each ROI were compared between conditions. Pain-evoked NPS expression (Wager *et al.*, 2013) was calculated to quantify changes in nociceptive activity between languages. We hypothesized that ‘language as inducer’ effects would generate higher intensity ratings, preceded by greater NPS expression, in the language of participants’ stronger cultural orientation. Alternatively, larger pain-evoked responses in the semantic or attention ROIs could suggest ‘language as meddler’ or ‘spotlight’ mechanisms for altered ratings of equivalent painful stimuli between languages.

## Methods

Complete methods available in Supplementary Materials: inclusion/exclusion criteria, stimuli and surveys, experimental protocol, fMRI scanning and preprocessing procedures, ROI construction, calculation of NPS and ROI activity.

### Participants

Thirty-nine (21 female) Spanish-English bilingual adults, recruited from the University of Miami or surrounding Miami-Dade County, ranging in age from 18 to 44 years (Mean ± SD 25.43 ± 5.74), comprise the present sample. Participants self-identified as Hispanic and/or Latino/a, fluently spoke both English and Spanish, had normal or corrected-to-normal vision and were compensated with money and/or course credit. Twenty-three participants were born in the USA while 16 immigrated to the USA at an average age of 10.89 ± 8.22 years. Twenty participants began in the English condition; 19 were counterbalanced to begin in Spanish. All procedures were conducted by one bilingual investigator and a bilingual MRI technician. The study protocol was approved by the University of Miami institutional review board, and participants provided written informed consent.

### Behavioral data analysis

Unless noted, all data were compiled and analyzed in R Studio version 3.6.1 (R Core Team, 2019).

Cultural orientation scores, on a –3 (completely Hispanic) to +3 (completely US-American) scale, were calculated for each participant based on Gianola *et al.*, 2021 (see supplement). The sample showed generally ‘balanced’ orientation (Mean ± SD 0.16 ± 0.44), ranging from –1.08 to +1.22 which did not vary between languages (*t*(39)= 1.09, *P* = 0.281, paired *t*-test).

Linear mixed regression models tested for effects of language and cultural orientation on pain intensity and unpleasantness ratings. Covariates included trial number, temperature, skin site, functional run, age, gender and counterbalance (i.e. English-first or Spanish-first). Random intercepts for participants accounted for variable pain sensitivity across individuals. Predictors of interest included language condition, cultural orientation and their interaction, with Satterthwaite’s degrees of freedom approximation (*lmerTest* package) used to assess significance of these variables’ influence on pain ratings. Cook’s distance was calculated (*Influence.ME* package) to identify potential participant-level and trial-level outliers. After checking for data entry or procedural errors, potentially influential observations were retained, consistent with statistical best practices for outlier analysis (Zuur *et al.*, 2010). Unless otherwise noted, reported results remain significant after excluding outliers and controlling for multiple comparisons across tests at false discovery rate (FDR) *q *< 0.05.

### fMRI data analysis

After preprocessing, univariate General Linear Models were constructed from first-level (individual run) neural data using FSL 5.0.9. All task components outside the baseline periods (i.e. instructions, image viewing, washout stimulation, between trial fixations, pain ratings) were modeled. The primary period of interest was the combined heat stimulation and fixed post-heat period (capturing subjective pain lingering) for each trial. First-level analysis identified BOLD responses significantly predicted by heat stimulations, controlling for motion, other task components and temporal derivatives. First-level maps were averaged across four functional runs per participant with language (English or Spanish) as a second-level covariate. Group analyses combined data across participants, controlling for counterbalance order and including a cultural orientation score covariate. This approach identified regions at a cluster-forming threshold of *Z *>2.3, *P *< 0.05 showing significant pain-evoked (i) general activity, (ii) activity differing across languages, (iii) activity correlating with cultural orientation and (iv) activity exhibiting a language by cultural orientation interaction. Group-level *Z* statistic maps were subjected to FDR correction for multiple comparisons, controlling the expected proportion of false-positive voxels (Benjamini and Hochberg, 1995; Genovese *et al.*, 2002) to *q *<0.05.

**Fig. 1.**
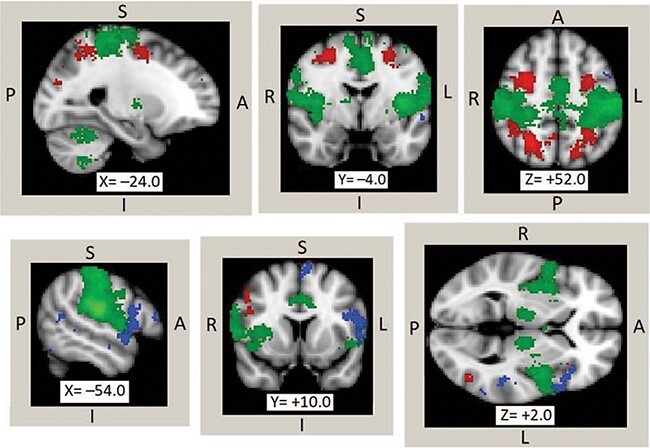
Neural maps of voxels in language-responsive/semantic (blue), attention (red) and somatosensory (green) regions of interest overlayed on standard MNI-152 template.

Procedures defining the semantic, somatosensory and attention ROIs (Figure 1), measuring their average BOLD responses and calculating NPS expression are described in the supplement. Trial-level NPS expression and average pain-evoked BOLD activity for each ROI were treated as outcomes in mixed effects regression models parallel to the explicit pain rating models (see ‘Behavioral Data Analysis’) with fixed effects for control variables, language condition, cultural orientation and their interaction and random intercepts across participants. These models tested whether pain-evoked activity in each ROI or NPS expression differed significantly across language conditions and/or cultural orientations.

**Fig. 2.**
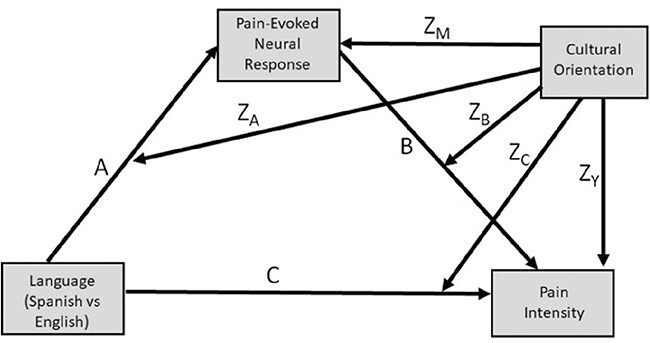
Moderated mediation pathway investigated with these analyses. ‘Pain-evoked neural response’ represents all tested mediators: NPS response, semantic ROI, attention ROI, and somatosensory ROI.

Follow-up moderated mediation analyses tested whether each neural indicator (i.e. NPS, somatosensory, attention and semantic ROIs) indirectly contributed to language effects on intensity ratings. Mediation models were consistent with the temporal precedence between pain-evoked neural responses (during stimulations) and pain ratings (post-stimulation). Mixed effects control models with random intercepts across participants and fixed effects for control variables (i.e. run, trial number, skin site, temperature, counterbalance, age, gender) were fit to intensity and neural mediator outcomes using the *lmer* function (Bates *et al.*, 2015). The residuals from these models were analyzed according to the moderated mediation pathway outlined in Figure 2. Residual-based mediation models were analyzed due to the data’s multilevel structure (trials within participants).

Mediation effects were compared at one standard deviation above (+0.60) and below (–0.28) the sample mean cultural orientation (+0.16), typical for testing conditional mediation effects (Preacher *et al.*, 2007). Path coefficients were estimated using *lm()* linear regression models (R Core Team, 2019) and corroborated against path models fit via *lavaan* package (Rosseel, 2012). Conditional mediation and direct effects were compared across moderator conditions (i.e. US-American and Hispanic cultural orientation) using the *mediation* package’s *test.modmed* function (Tingley *et al.*, 2014). Bootstrapped confidence intervals were calculated from 1000 simulations per conditional model and 2000 simulations for comparisons across conditional models. These models were interpreted according to the mediation framework outlined by Zhao *et al.* (2010).

## Results

### Language effects on behavioral outcomes

**Fig. 3.**
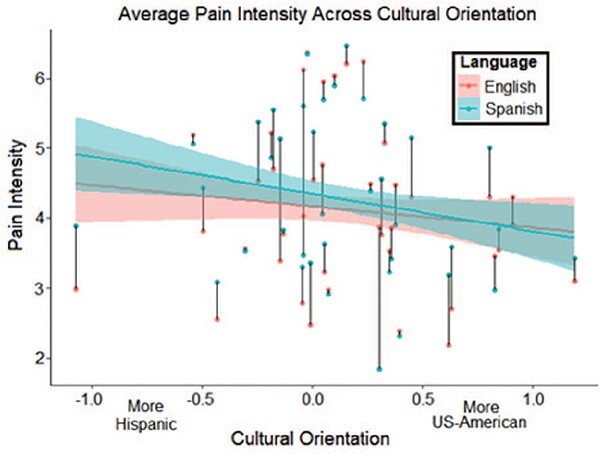
Plotted relationship between cultural orientation scores and average pain intensity within each language condition. The intensity rating scale ranged from 0 to 10. Black lines connect dots representing each participant’s average pain response across all trials in the English (warm colors, red dots) or Spanish (cool color, blue dots) condition. Colored lines with error bands show the overall effect across cultural orientation in each language. Negative cultural orientation values represent greater endorsement of Hispanic compared to US-American culture; positive values reflect the opposite; scores near zero correspond to balanced cultural orientation. Error bands denote standard error of the mean.

Overall participants rated heat stimulations delivered during Spanish runs as 0.21 points more intense (0–10 scale) compared to English runs (β = 0.21, *t*(1361)= 2.88, *P *= 0.004; Figure 3). This language effect on intensity ratings (Cohen’s *d *= 0.16) was comparable to intensity reductions for stimulations delivered to the least sensitive skin site (*d *= –0.18). Though not significant, the language by cultural orientation interaction on intensity ratings trended in the same direction (β = –0.26, *P *= 0.102) as observed previously (Gianola *et al.*, 2021), with reduced effect size (*d *= –0.09 *vs d *= –0.14 previously). This interaction effect did reach significance when excluding 34 outlier trials (2.43% of sample; β = –0.34, *P*= 0.019). In total, fixed effects (i.e. control variables, language, cultural orientation and their interaction) accounted for 47.3% of the variance in intensity ratings (adjusted R^2^). The full model with random intercepts across participants accounted for 69.5% of pain intensity variance.

Consistent with our prior findings (Gianola *et al.*, 2021), pain unpleasantness was not predicted by language condition, cultural orientation or their interaction (*P’*s >0.30). Fixed effects explained 42.2% of the variance in unpleasantness. Given the lack of significant interaction effects, more parsimonious models without the interaction were fit to these data, wherein the same pattern of results held (Table S3). For ease of comparison with neural outcomes, Table 1 summarizes results for predictors of interest including interactions.

**Table 1.** Fixed effects parameter estimates and statistical tests for predictors of interest across behavioral pain outcomes

Predictors	Slope	Standard Error	95% Confidence Interval	*P*-value
Intensity Ratings				
Language*	0.21	0.07	0.07 — 0.34	0.004
Cultural Orientation	–0.27	0.31	–0.27 — 0.32	0.373
Language by Cultural Orientation	–0.26	0.16	–0.57 — 0.05	0.102
Unpleasantness Ratings				
Language	–0.01	0.08	–0.16 — 0.14	0.861
Cultural Orientation	–0.32	0.34	–0.97 — 0.34	0.351
Language by Cultural Orientation	–0.04	0.17	–0.37 — 0.30	0.831

*Notes:* Positive language effects represent higher pain ratings in Spanish. Positive cultural orientation effects reflect higher pain ratings with increasing US-American orientation; *P* values rounded to three decimals.

* *q*< 0.05 FDR corrected for multiple comparisons across all tests of variables of interest.

### Whole brain pain-evoked activity

**Fig. 4.**
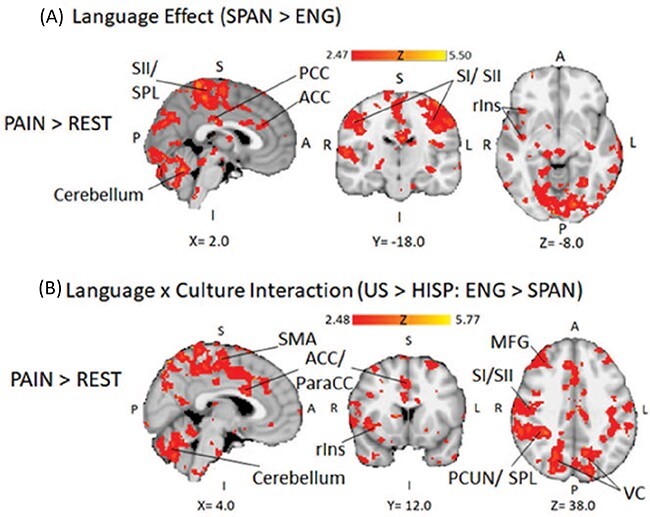
(A) Cross-section of whole-brain map overlayed on standard MNI-152 template highlighting regions showing increased BOLD activation in Spanish compared to English during heat stimulations. (B) Whole-brain map on MNI-152 template highlighting significant clusters which showed increases in pain-evoked BOLD activity in English compared to Spanish as a function of greater US-American (*vs* Hispanic) cultural orientation. Maps are FDR corrected for multiple comparisons at *q *< 0.01. Abbreviations: ACC, Anterior Cingulate Cortex; MFG, Middle Frontal Gyrus; ParaCC, Paracingulate Cortex; PCC, Posterior Cingulate Cortex; PCUN, Precuneus, rIns- right Insula; SI, Primary Somatosensory Cortex; SII, Secondary Somatosensory Cortex; SMA, Supplementary Motor Area; SPL, Superior Parietal Lobule; VC, Visual Cortex.

Cross-language contrasts of neural responses averaged across participants served to identify regions exhibiting modulated pain-evoked activity between languages. Consistent with intensity ratings, increased BOLD activation during painful heat in Spanish relative to English emerged within well-established contributors to the affective (anterior insula, ACC) and sensory (e.g. SI, SII, posterior cingulate and insula) processing of pain (Treede *et al.*, 1999; Morton *et al.*, 2016), and regions involved in attentional modulation of pain (left SPL, cerebellum; (Wiech *et al.*, 2005; Bushnell *et al.*, 2013). This contrast further showed greater insular activity contralateral to stimulation and more left-lateralized bilateral cerebellar activity, patterns expected from prior pain studies (Bingel *et al.*, 2002). Various regions showed cross-language pain-evoked activity difference at FDR corrected *q *< 0.01 (Figure 4A).

To investigate regions implicated in cultural orientation-moderated language effects on pain, a cross-level orientation by language contrast identified clusters whose pain-evoked BOLD activity in English increased (at FDR *q *< 0.01) with higher US-American cultural orientation. Various cortical regions showed increased bilateral activity associated with this interaction: supplementary motor area, ACC and paracingulate gyrus, SI/SII, precuneus extending into SPL and MFG. Prior research suggests greater attentional shifting (MFG) toward and maintained awareness (SPL) on the sensory (SI/SII, Ins) and affective (ACC) experience of the pain (Japee *et al.*, 2015; Morton *et al.*, 2016) in participants’ culturally preferred language could explain this activation pattern for this contrast. Though these areas showed bilateral activity increases, their spatial extents were larger contralateral to pain, while bilateral cerebellar activity showed a larger ipsilateral extent, both patterns associated with lateralized pain processing (Coghill *et al.*, 2001). Furthermore, right insula, opercular cortex and MTG exhibited higher pain-evoked activity in participant’s culturally preferred language, potentially suggesting greater online language processing (Friederici, 2011). In total, several regions exhibited increased BOLD activity during pain in a language congruent with participants’ stronger cultural orientation (Figure 4B).

### Language effects on neural outcomes moderated by cultural orientation

**Fig. 5.**
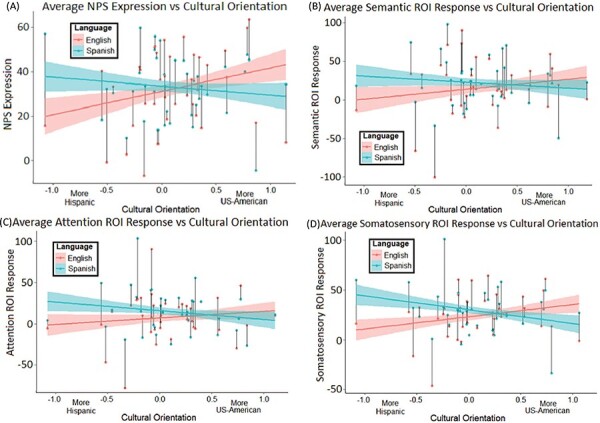
Plotted relationship between cultural orientation scores and average pain-evoked neural responses for (A) NPS expression, (B) semantic ROI, (C) attention ROI and (D) somatosensory ROI pain-evoked BOLD response. Y-axes represent arbitrary units relative to baseline activity (0-point). Black lines connect dots representing each participant’s average pain-evoked response across all trials in the English (warm, red) or Spanish (cool, blue) condition. Colored lines with error bands show the overall effect across cultural orientation in each language. Negative cultural orientation values represent greater endorsement of Hispanic compared to US-American culture; positive values reflect the opposite; scores near zero correspond to balanced cultural orientation. Error bands denote standard error.

Participants’ trial-level pain-evoked NPS responses differed across language conditions and cultural orientation patterns. Effects of language (β= 0.05, *t*(1317)= 2.00, *P *= 0.046) and cultural orientation (β= 0.16, *t*(86)= 2.24, *P *= 0.027) were seen to influence NPS expression, though these main effects are obscured by significant moderation of language effects by cultural orientation. That is, NPS expression was greater in the language of participants’ stronger cultural orientation (β = –0.16, *t*(1313) = –4.71, *P *< 0.001; Table 2, Figure 5A). Cohen’s d of –0.26 shows this interaction influenced NPS expression to a degree comparable to receiving stimulations on the least sensitive skin site (d = –0.30), one-third as large as increasing the stimulation temperature by 1ºC (d= 0.76). Culturally moderated language effects appear to contribute substantially to this nociceptive indicator. The fixed effects (i.e. control variables, language, cultural orientation and their interaction) accounted for 17.3% of NPS variance (34.8% explained by full model).

**Table 2.** Fixed effects parameter estimates and statistical tests for predictors of interest across neural pain outcomes

Predictors	Slope	Standard Error	95% Confidence Interval	*P*-value
Semantic ROI BOLD Response				
Language*	0.08	0.03	0.03 — 0.13	0.001
Cultural Orientation*	0.19	0.08	0.03 — 0.34	0.018
Language by Cultural Orientation*	–0.13	0.04	–0.20 — −0.06	0.001
Attention ROI BOLD Response				
Language*	0.16	0.03	0.11 — 0.21	<0.001
Cultural Orientation*	0.22	0.07	0.08 — 0.37	0.003
Language by Cultural Orientation*	–0.22	0.04	–0.29 — –0.14	<0.001
Somatosensory ROI BOLD Response				
Language*	0.15	0.03	0.10 — 0.20	<0.001
Cultural Orientation*	0.22	0.07	0.08 — 0.35	0.002
Language by Cultural Orientation*	–0.26	0.04	–0.33 — –0.18	<0.001
NPS Response		0.02	0.00 — 0.10	
Language**^†^**	0.05			0.046
Cultural Orientation*	0.16	0.07	0.02 — 0.29	0.027
Language by Cultural Orientation*	–0.16	0.03	–0.23 — –0.10	<0.001

*Notes:* Model outcomes for predictors of interest in pain-evoked neural responses. Positive language effects represent higher pain-evoked activity in Spanish. All estimates are standardized and rounded to two decimals.

*P* values rounded to three decimals. *q< 0.05 FDR corrected for multiple comparisons across all tests of interest.

† *P *< 0.05 but *q *> 0.05 after FDR correction.

Our tested ROIs exhibited similar language moderated cultural orientation effects in their pain-evoked activity. Main effects for language and cultural orientation could indicate higher pain-evoked activity in Spanish and among more US-American oriented participants. However, significant interactions between language and cultural orientation reveal that participants’ pain-evoked activity in ROIs related to semantic (β = –0.13, *t*(1312) = –3.48, *P *= 0.001), attention (β = –0.22, *t*(1313) = —5.85, *P *< 0.001) and somatosensory processing (β = –0.26, *t*(1315) = –6.99, *P *< 0.001) were higher in the language congruent with their stronger cultural orientation (Table 2, Figure 5B-D). This culturally moderated language effect in the attention ROI (d = –0.32) was nearly twice as large as a 1ºC increase in stimulation temperature (d = 0.17), implying that cultural salience affected participants’ attention more than stimulus intensity. The somatosensory ROI showed a similar effect size (d = –0.39), compared to a weaker effect for the semantic ROI (d = –0.19), both of which approached the magnitude of a 1ºC increase in each region (d = 0.50, d = 0.31, respectively). These models’ fixed effects explained 7.2% of pain-evoked activity variance in semantic ROI, 9.1% in attention ROI, and 13.7% in somatosensory ROI (variance explained by full models: 28.9%, 27.2% and 27.6%, respectively).

### Moderated mediation of language effects by NPS and somatosensory responses

**Fig. 6.**
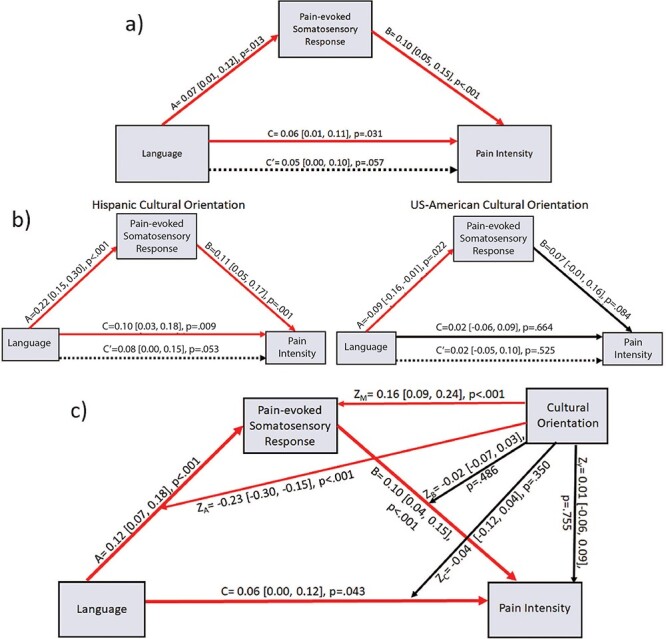
Path diagrams for language to pain intensity relationship with pain-evoked somatosensory ROI activity as mediating variable. (A) Direct mediation diagram across the full sample; (B) conditional mediation diagrams for Hispanic oriented (left) and US-American oriented (right) participants; (C) full moderated mediation diagram, including cultural orientation effects on all paths. Red arrows denote significant (*P *< 0.05) paths.

**Fig. 7.**
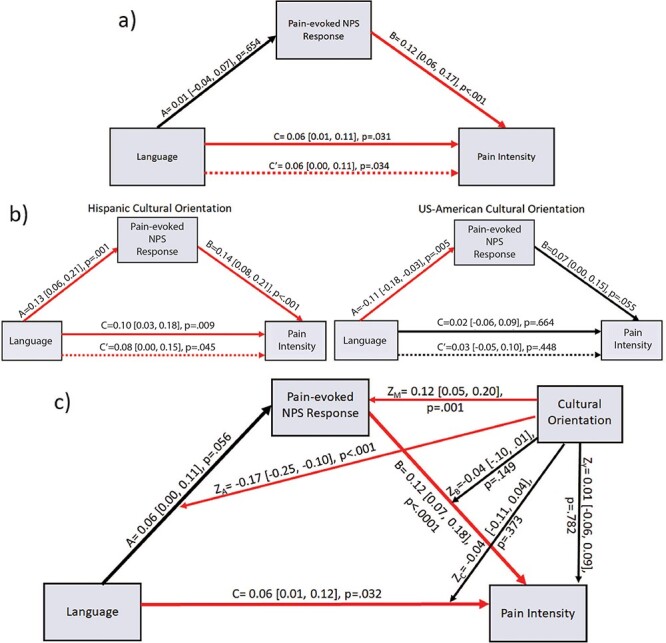
Path diagrams for language to pain intensity relationship with pain-evoked NPS expression as mediating variable. (A) Direct mediation diagram across the full sample; (B) conditional mediation diagrams for Hispanic oriented (left) and US-American oriented (right) participants; (C) full moderated mediation diagram, including cultural orientation effects. Red arrows denote significant (*P* < 0.05) paths.

Across low variance inflation factors trials (*n* = 1353, see supplement), language effects on pain intensity were mediated by average pain-evoked BOLD activity in somatosensory regions. Furthermore, the strength and direction of mediation effects for both NPS and somatosensory activity differed significantly across cultural orientation levels. Specifically, NPS and somatosensory responses positively mediated the language—pain intensity relationship among Hispanic oriented participants while more US-American oriented participants showed weaker negative mediation effects. We describe moderated mediation models for somatosensory ROI (Figure 6) and NPS (Figure 7) responses in turn.

Across participants, pain-evoked somatosensory ROI activity significantly mediated language effects on pain intensity. In Spanish, overall somatosensory responses (path *a* = 0.07, 95% CI [0.01, 0.12], *P* = 0.013) and pain intensity ratings (path *c* = 0.06, 95% CI [0.01, 0.11], *P *= 0.031) were higher compared to English. As pain-evoked somatosensory ROI activity, controlling for language, also predicted intensity ratings (path *b* = 0.10, 95% CI [0.05, 0.15], *P *< 0.001), a significant mediation effect (path *ab* = 0.016, 95% CI [0.003, 0.030], *P *= 0.022) was observed. From the reduction in language to intensity effects (path *c’* = 0.05, 95% CI [0.00, 0.10], *P *= 0.057), we found that pain-evoked somatosensory ROI responses mediated 11.3% of total effects (Figure 6A).

Moderating effects of cultural orientation clarify that this mediation was largely driven by Hispanic oriented participants. That is, among participants reporting Hispanic orientation, language condition strongly predicted both pain-evoked somatosensory ROI activity (path *a *= 0.22, 95% CI [0.15, 0.30], *P *< 0.001) and intensity ratings (path *c *= 0.10, 95% CI [0.03, 0.18], *P *= 0.009). Somatosensory responses in turn predicted intensity ratings, controlling for language (path *b *= 0.11, 95% CI [0.05, 0.17], *P *< 0.001), resulting in a significant mediation effect (path *ab *= 0.06, 95% CI [0.02, 0.11], *P *= 0.004). The direct effect (path *c’ *= 0.08, 95% CI [0.00, 0.15], *P*= 0.053) of language on intensity ratings is reduced relative to the total effect, with 24.2% of this relationship mediated by somatosensory activity for Hispanic oriented participants (Figure 6B left). Conversely, this mediation pathway trended marginally in the opposite direction among more US-American orientated participants (path *ab *= –0.015, 95% CI [–0.04, 0.00], *P *= 0.078). Weaker mediation was driven by lower pain-evoked somatosensory responses in Spanish relative to English (path *a *= –0.09, 95% CI [–0.16, –0.01], *P *= 0.022) and marginal prediction of pain intensity by somatosensory activity (path *b*= 0.07, 95% CI [–0.01, 0.16], *P *= 0.084). More US-American oriented participants did not show significant total or direct effects of language on intensity ratings (*c* and *c’* paths *P*’s >0.50; Figure 6B right). These inverse mediation effects were thus significantly different across cultural orientations (*ab*_Hisp_−*ab*_US_ = 0.07, 95% CI [0.03, 0.12], *P* = .001), while we lack sufficient evidence to confirm stronger direct effects of language on intensity among Hispanic relative to US-American oriented participants (*c’*_Hisp_—*c’*_US_ = 0.12, 95% CI [–0.13, 0.37], *P* = .327). Including cultural orientation in the model shows 15.1% of language effects on intensity mediated by somatosensory ROI responses across participants (Figure 6C).

Unlike somatosensory ROI activity, NPS expression did not mediate language to pain intensity effects before accounting for cultural orientation. Although language condition significantly predicted intensity ratings across the sample (path *c *= 0.06, 95% CI [0.01, 0.11], *P *= 0.031), inverse language effects on NPS responses between Hispanic and US-American oriented participants resulted in non-significant prediction of pain-evoked NPS by language (path *a *= 0.01, 95% CI [–0.04, 0.07], *P *= 0.654). Despite the positive relationship between NPS and intensity ratings (path *b *= 0.12, 95% CI [0.06, 0.17], *P *< 0.001), the mediation remained non-significant (path *ab *= 0.004, 95% CI [–0.01, 0.02], *P *= 0.632) while direct effects of language on intensity (path *c’ *= 0.06, 95% CI [0.00, 0.11], *P *= 0.034) were equivalent to total effects (Figure 7A).

Including cultural orientation, NPS expression partially mediated the language-intensity relationship among Hispanic oriented participants. Participants reporting higher Hispanic orientation showed larger NPS responses in Spanish (path *a *= 0.13, 95% CI [0.06, 0.21], *P *< 0.001) in turn predicting higher intensity ratings (path *b *= 0.14, 95% CI [0.08, 0.21], *P *< 0.001), resulting in significant mediation (path *ab *= 0.05, 95% CI [0.02, 0.08], *P *< 0.001). Therefore, these participants’ direct language effects on intensity (path *c’ *= 0.08, 95% CI [0.00, 0.15], *P *= 0.045) were reduced relative to total effects (path *c *= 0.10, 95% CI [0.03, 0.18], *P *= 0.009), with 19.6% mediated by NPS expression (Figure 7B left). Conversely, US-American oriented participants produced lower NPS responses in Spanish (path *a *= –0.11, 95% CI [–0.18,—0.03], *P *= 0.005), while NPS signal only marginally predicted intensity ratings (path *b *= 0.07, 95% CI [0.00, 0.15], *P *= 0.055), producing a marginal indirect effect (path *ab *= –0.02, 95% CI [–0.05, 0.00], *P *= 0.066) in the opposite direction. Neither direct nor total effects of language on intensity ratings were significant (*P*’s > 0.40), representing indirect-only mediation among US-American oriented participants (Zhao *et al.*, 2010). Inverse NPS-mediated language effects on intensity thus differed significantly between US-American and Hispanic oriented participants (*ab*_Hisp_−*ab*_US_ = 0.06, 95% CI [0.03, 0.11], *P* < .001) while direct language-intensity effects did not (*c’*_Hisp_—*c’*_US_ = 0.12, 95% CI [–0.14, 0.36], *P* = .378). Accounting for moderation by cultural orientation, NPS expression mediated an estimated 9.4% of total language effects on intensity ratings (Figure 7C).

## Discussion

This study investigated whether pain experiences are shaped by language and cultural contexts and the neurobiology underlying this relationship. Spanish–English bilingual adults rated painful heat in separate English and Spanish contexts during fMRI. Both explicit pain ratings and pain-evoked neural activity were sensitive to language conditions. Specifically, participants produced larger intensity ratings in Spanish overall while average BOLD responses in regions implicated in attention and semantic processing showed larger pain-evoked increases in participants’ culturally preferred language. Moreover, direct sensory processing of pain (represented via somatosensory ROI and NPS responses) mediated the relationship between the language context and pain intensity. We observed meaningful changes in neural processing and reported experiences of pain between languages.

### Behavioral and whole brain analyses

Language effects on intensity (but not unpleasantness) ratings replicate our earlier findings (Gianola *et al.*, 2021) while implying cultural contexts may exert greater influence on sensory-discriminative (*vs* affective) aspects of pain (Auvray *et al.*, 2010). We observed higher pain-evoked activity for Spanish overall in somatosensory (SI/SII), posterior and mid-cingulate cortex, cerebellum and posterior insula (Figure 4A), with wider extent contralateral to stimulation. This pattern, which also emerged during pain delivered in English among more US-American oriented participants (Figure 4B), is suggestive of sensory-discriminative nociceptive processes (Maihöfner *et al.*, 2006; Segerdahl *et al.*, 2015), as these portions of cingulate cortex preferentially activate during pain compared to cognitive or emotional tasks (Kragel *et al.*, 2018). Though pain context can affect both intensity and unpleasantness judgments, explicit focus on (Moseley and Arntz, 2007) or distraction from pain (Kenntner-Mabiala *et al.*, 2008) can selectively alter intensity judgments without unpleasantness changes. Differences in directed attention may explain why Hispanic orientated participants (who mostly learned Spanish first) showed larger intensity reductions in English, as lower proficiency entails greater effort during second language processing (Pivneva *et al.*, 2012). Distraction effects within the less dominant language are further implied by increased activity in regions associated with reorienting awareness and attentional pain modulation (SPL, MFG) in participant’s culturally preferred language (Bushnell *et al.*, 2013; Japee *et al.*, 2015). In total, altered sensory-discriminative pain processing may account for different intensity report between languages, while reduced pain attention in a less preferred language may contribute to a trend toward language by cultural orientation interaction.

### ROI and NPS neural analysis

This investigation augments work examining functional organization of language influenced cognition (Athanasopoulos and Casaponsa, 2020) by elucidating neural mechanisms of language effects on bilingual pain. As cultural orientation significantly moderated language effects on pain-evoked NPS responses, a validated metric of nociceptive activity (Wager *et al.*, 2013; Zunhammer *et al.*, 2018; Han *et al.*, 2022), bilingual adults’ nociceptive responses to noxious heat increased in the language of their stronger cultural orientation. The multivariate NPS pattern exhibits excellent short-term test-retest reliability for within-person effects (Han *et al.*, 2022) and is largely unaffected by placebo analgesia manipulations, even those affecting explicit pain report (Zunhammer *et al.*, 2018). Thus, rather than representing expectation or conditioning placebo effects (Schafer *et al.*, 2018), this finding reinforces the language-as-inducer hypothesis, as not just pain ratings, but underlying nociceptive neural activity, varied across bilingual adults’ language contexts.

The univariate ROI analyses complement these results, implying that somatosensory areas contribute to altered nociceptive processing between languages. For average pain-evoked somatosensory ROI activity, the same language by culture interaction was larger (relative to NPS expression) and directly mediated the language effect on intensity ratings. The moderated mediation of both somatosensory ROI and NPS responses by cultural orientation clarifies why cross-language pain intensity differences were largest among Hispanic oriented participants. While language predicted pain-evoked NPS and somatosensory ROI responses at both ends of the cultural orientation spectrum, greater activity only translated into higher intensity ratings among Hispanic oriented participants. This result mirrors previous findings that self-reported ethnic identification correlates with experimental cold and heat pain responses among Hispanic (not non-Hispanic) participants (Rahim-Williams *et al.*, 2007). Thus, how strongly a schematic mode, affecting nociceptive and somatosensory sensitivity to pain, is invoked by English or Spanish may depend on individuals’ connection to the cultural frames each language represents.

Beyond predicting explicit intensity, language as ‘spotlight’ or ‘meddler’ hypotheses may refine our understanding of cross-language variation in pain-evoked activity. Besides perhaps inhibitory motor control associated with IFG (Swick *et al.*, 2008), the left-lateralized areas comprising the semantic ROI were not expected to exhibit pain-evoked activity, particularly ipsilaterally (Boisgueheneuc *et al.*, 2006; Costafreda *et al.*, 2006; Friederici, 2011; Alagapan *et al.*, 2019). Therefore, above-baseline pain-evoked semantic ROI responses showing consistent ‘differences’ between languages could reflect increased tendency to internally describe the pain in one’s culturally preferred language. Larger attention ROI responses in participants’ culturally preferred language could similarly imply greater salience afforded to the sensory experience in one’s preferred cultural context. However, reverse inference interpretations require first corroborating this pattern across languages via experimental attention manipulations during pain. Language effects for semantic and attention ROIs were largest among Hispanic oriented participants, with weaker inverse effects among US-American oriented participants. Perhaps, both the meddling of linguistic codes and the spotlight those codes place on specific aspects of pain experiences mutually reinforced each other among participants that felt greater distinctions across cultural contexts. Overall, these analyses illuminate relevant mechanisms through which language may affect pain, as moderated by cultural orientation.

### Connections to Hispanic health

This study connecting cultural context and pain may hold relevance for pain assessment and treatment across Hispanic populations, while the ‘Hispanic paradox’ framework further clarifies our results. Coupled with knowledge that social information can alter responses to experimental pain (Koban and Wager, 2016) and language concordant care generally improves patient satisfaction and compliance (Ali and Johnson, 2017), our findings indicate that language selection during pain care should integrate patients’ cultural profiles with the goal of communication (e.g. accurate assessment *vs* pain distraction). Lower pain report (and reduced somatosensory activity) in English among Hispanic oriented participants could contribute to lower likelihood to receive adequate pain treatment among Hispanics (Meghani *et al.*, 2012; Lee *et al.*, 2019); clinicians limited to English may receive pain reports dampened relative to patients’ internal experiences. While medical Spanish training is increasingly integrated in medical schools (Ortega *et al.*, 2021), our results reinforce calls to incorporate assessment methodology and post-course competency evaluations in language training (Ortega *et al.*, 2019, 2020). When preferred-language providers are unavailable, translation during pain assessment and treatment, especially from individuals who share the patient’s cultural frame, may improve outcomes. Participants’ higher intensity ratings in Spanish bolster claims that linguistic features of Spanish impact emotional and evaluative processes (Llabre, 2021). Culturally informed stress appraisals, theorized to buffer against negative health outcomes (Ruiz *et al.*, 2018), may explain why larger neural pain responses did not translate into greater unpleasantness. Moreover, US acculturation among Puerto Ricans is hypothesized to explain greater morbidity for chronic pain (relative to other Hispanic backgrounds) due to less protective psychosocial factors (Nahin, 2021). Decreased nociceptive and somatosensory activity associated with Hispanic orientation might clarify Nahin’s results, particularly for bilingual patients assessed in English. Nevertheless, language effects must not overshadow deeper inequities in pain assessment and treatment (K. O. Anderson *et al.*, 2009; Lee *et al.*, 2019), as improved assessment will not resolve Hispanic patients’ reduced likelihood to have their pain assessed at all (Meghani *et al.*, 2012). In conclusion, variability in health outcomes within and across ethnicities labeled ‘Hispanic’ may be influenced by culturally informed behaviors and attitudes varying across language contexts.

### Limitations and future directions

Strong interpretation of these findings must be tempered by pertinent limitations of our sample, experimental design and analysis. First, low sample size restricted our ability to detect weaker language or cultural effects, likely contributing to weaker interaction effects for intensity ratings (Gianola *et al.*, 2021). Our early bilingual sample (both languages acquired by age 10) was not differentiated between compound (languages learned together) and coordinate bilingualism (one language learned first), a factor linked to language use and cultural identity (Huynh *et al.*, 2011), potentially influencing the uneven distribution of cultural orientations. Completing both language conditions in one day may increase the salience of contrast between the Spanish condition and US university environment. As ethnic identities in minoritized contexts become more salient (Umaña-Taylor and Shin, 2007), the experimental environment may have been less stressful for participants preferring US-American culture, dampening cross-language pain differences, as lower stress relates to decreased pain sensitivity (Mertens *et al.*, 2020). Furthermore, differences in pain outcomes observed here cannot be attributed to language (Spanish *vs* English) context specifically, as language and cultural priming (Hispanic *vs* US-American) manipulations were combined, preventing dissociation of their effects (Yoo *et al.*, 2014). Though qualified by these limitations, our analyses shed crucial light on when and how language can influence pain perception.

Our experimental paradigm and analyses should be extended to address related research questions. Replicating the present study across separate English and Spanish scanning days, with heat stimulations delivered before and after language-congruent cultural priming, could disentangle relative contributions of environment, language and cultural priming on pain. Having found evidence for language-as-meddler effects, we can generate hypotheses about specific lexical/grammatic differences in expressing bodily sensations between languages (e.g. diminutive forms, ‘be hot’ *vs tener calor—*‘have heat’) to design experiments focused on those linguistic structures. This paradigm should be adapted across different populations: comparing Spanish and English monolinguals, bilingual chronic pain patients, bi- or multi-linguals speakers of more linguistically distinct languages (e.g. English–Korean, Spanish–Turkish). This study offers a basis to elucidate neural mechanisms underlying connections between language, culture and pain.

## Conclusion

We explored whether and how momentary cultural/language context influences processing and perception of acute pain among Spanish–English bilingual adults. Participants reported higher pain intensity in Spanish while exhibiting relatively higher pain-evoked neural activity in the language associated with their stronger cultural orientation. Cross-language activity differences emerged in selected brain-based indicators of nociceptive, somatosensory, attention and semantic processing; somatosensory and nociceptive processing indicators mediated the relationship between language and pain intensity for Hispanic oriented participants. This investigation contributes to our understanding of how culture and language may shape perception at neural and cognitive levels.

## Supplementary Material

nsad074_Supp

## Data Availability

All relevant data and analysis code for this project are available via Open Science Framework: https://osf.io/8djb7/?view_only=20c31cf31785432289cd1d7fd7cc39db.

## Footnotes

1 While long-standing debates exist around the terms chosen to describe the diaspora of peoples originating from Spanish-speaking or Spanish-influenced countries (Hayes-Bautista and Chapa, 1987; Salinas, 2020), they are not the focus of this article. The term ‘Hispanic’, frequently incorporated in the sociocultural and health literature and with which the majority of our sample self-identified, is employed throughout this text.
