# Supplementary material for: Does pain hurt more in Spanish? The neurobiology of pain among Spanish–English bilingual adults
Source: Soc Cogn Affect Neurosci. 2023 Dec 15;19(1):nsad074. doi: 10.1093/scan/nsad074 (PMC10868134; doi:10.1093/scan/nsad074)
Supplement: nsad074_Supp [file nsad074_supp.zip › scan-23-126-File011.docx]

**SUPPLEMENTARY MATERIAL:**

**Supplemental Methods:**

*Participants:*

We incorporated the following inclusion and exclusion criteria within our sample collection. Individuals under age 18 were excluded due to ethical concerns with administering painful stimulation to minors, while those over 55 were excluded due to documented alterations in pain sensitivity in this age group (Riley 3rd et al., 2010). Individuals with current or past mental disorder or nervous system disease, claustrophobia, MRI contraindications (e.g., metal in the body), pain or illness on the scan day, or taking medications known to alter pain processing were further excluded. Of the 42 participants initially recruited two participants (one of whom only completed half the MRI scan) showed excessive head motion throughout scanning and were removed from all behavioral and neural analyses. One further participant was excluded from pain task analyses, as she showed an extreme bias in pain ratings across languages (near 0 in Spanish trials) and represented a highly influential outlier in our models. She will be the focus of a separate single-subject analysis.

Most participants reported learning Spanish before English, beginning to learn Spanish by 1.51 ± 1.24 years (Mean ± SD) and acquiring fluency by 4.13 ± 1.99 years, while they began learning English by age 4.23 ± 2.37 and reached fluency by 6.71 ± 4.05 years. All participants therefore had significant contact with both languages by age 10, which is often used as a cutoff for early vs late bilingualism (Luk et al., 2011).

*Stimuli and Survey Measures:*

All stimuli viewed by participants during training and inside the scanner were delivered via an experiment using Presentation® software (Version 20.0, Neurobehavioral Systems, Inc., Berkeley, CA). The cultural priming images shown during training and the pain rating task were selected from images characterized in an online survey study of Spanish-English bilingual adults (Gianola et al., 2020). The same nine US-American images were shown during two runs of the English pain rating task, and nine Hispanic images were shown during the two Spanish runs. To maximize the cognitive effect of viewing these cultural primes in the scanner, distinct images from those seen in the scanner were shown during the training (see Experimental Procedure).

Sentence stimuli for the semantic judgement task were adapted from a corpus of 156 English sentences, half of which were semantically plausible (e.g. “Bulls charge with great ferocity”) and half semantically implausible (e.g., “Kangaroos type for their jobs”), previously used in a study of reading fluency (Christodoulou et al., 2014). A subset of 32 sentences for the fMRI task and 16 sentences for the training procedures were selected, half of each set being semantically plausible. Half of both plausible and implausible sentences were written in the passive voice (e.g., “the cake was baked”) while the remainder had active constructions (e.g., “the man baked the cake”). The 48 selected sentences were translated to Spanish and back translated to English by separate bilingual individuals, with any discrepancies between the original content and back translations resolved by discussion among the translators, in line with recommended best practices for translating study materials (Prieto, 1992). The sentences used during task training were distinct from those seen in the scanner.

Painful thermal stimulations were administered using an MRI-safe 16mm x 16mm Medoc contact thermode placed against the participant’s left volar forearm. “Suprathreshold” heat stimulations were delivered for a sustained period at one of three set temperatures (46°C, 47°C, 48°C) for each pain trial. These temperatures have been identified in previous studies as above most individuals’ pain threshold (Edwards & Fillingim, 1999). Each heat stimulation lasted 8 seconds in total, comprising 4.6 seconds at the target temperature flanked by 1.7 second ramp periods to get to/from the target temperature from/to the 32ºC baseline. Four different skin sites on the forearm were stimulated over the course of the study to control for individual differences in local skin pain sensitivity and to minimize the duration of heat delivered to any one area. After each stimulation, participants provided ratings of their overall pain intensity and unpleasantness by rating with a mouse on a 0-10 visual rating scale (0 = “No Pain”/ “No Unpleasantness”, 10 = “Worst Pain Imaginable” / “Worst Unpleasantness Imaginable”). In the Spanish condition, the anchors on the rating scales read from 0= “Nada de Dolor”/ “Nada Desagradable” to 10= “El Peor Dolor Imaginable”/ “Lo Más Desagradable Imagainable”. Prior work has related ratings of intensity to the sensory-discriminative aspects of pain, while unpleasantness ratings reflect the affective-motivational aspects of pain (Coghill et al., 1999).

In addition to a short recruitment survey used to screen community members for study eligibility, participants completed self-report surveys administered once each in English and Spanish. Surveys included the Big Five Inventory of personality (John & Srivastava, 1999), the Abbreviated Multidimensional Acculturation Scale (Zea et al., 2003), the Sociocultural Questionnaire from the Hispanic Community Health Study (Sorlie et al., 2010), the Bicultural Involvement Questionnaire (Szapocznik et al., 1980), the Bicultural Identity Integration Scale-Version 2 (Benet‐Martínez & Haritatos, 2005), and the Language Experience and Proficiency Questionnaire (Marian et al., 2007). Comparative socioeconomic status across the sample was recorded via the Barratt Simplified Measure of Social Status (Barratt, 2006). Overall, socioeconomic status did not vary significantly across participant gender or assigned counterbalance order when completing this survey in either language (all p’s >.15, two-sample *t*-tests), nor did they vary across survey languages (p= .79, paired t-test). Two participants did not provide socioeconomic information in either language, and one participant only provided this information in Spanish. All other surveys were completed by all participants in both languages. Finally, participants were administered an in-house completion survey which assessed their beliefs about the study goals and in which they rated aspects of the cultural priming images. The completion survey was the only measure not completed in both languages.

Following the analysis of the precursor to this study (Gianola et al., 2021), participants’ relative orientation toward US-American and Hispanic culture was calculated via a composite measure of items from the Abbreviated Multidimensional Acculturation Scale (AMAS) which quantifies degree of identification with both US-American culture and “culture of origin” (Zea et al., 2003) and the Sociocultural Questionnaire from the Hispanic Community Health Study/Study of Latinos (SOL-SC) which includes questions covering themes of language use, familism, religion, and perceived discrimination (Sorlie et al., 2010). Cultural orientation scores were calculated in the following manner: AMAS culture of origin subscale was subtracted from the US culture subscale, rescaled, and combined with the acculturation and reverse scored familism subscales of the SOL-SC via a weighted average. This produced a single continuous metric theoretically ranging from –3 (total endorsement of Hispanic culture, no endorsement of US-American culture) to +3 (opposite cultural profile), with 0 representing a balanced bicultural orientation.

*Experimental Procedure:*

Unless otherwise indicated, all participant interactions described here were conducted by the author (MG) who is fluent in both English and Spanish. Potential study participants provided information regarding their eligibility and preferred form of contact in an online screening survey managed by REDcap. Individuals who appeared to meet eligibility criteria were contacted and further screened for eligibility, including conversational competence in both English and Spanish. If they met all criteria, they were scheduled for a single in-laboratory study session. Before their session, participants were counterbalanced to either the English-first or Spanish-first condition, which determined their training language and order of in-scanner functional runs (English vs Spanish).

Written informed consent was obtained from all participants in English at the beginning of their study session. After consent, all written and verbal communication switched to Spanish for Spanish-first counterbalanced participants, as they completed MRI screening forms (all procedures for English-first counterbalanced participants were equivalent to those described here except for the language order). The participant was taken to the experiment room where they completed a series of surveys in Spanish (see *Stimuli and Survey Measures*). The experimenter then trained the participant on the two study tasks: semantic judgement and pain rating. The participant completed a shortened version of the semantic judgement task with written and spoken instructions and two examples of plausible and implausible sentences. The experimenter then explained the pain rating scales and carefully outlined the difference between pain intensity and unpleasantness using previously developed language (Price et al., 1983). The participant received four heat stimulations (of 46, 47, 48, and 47°C) intermixed between four Hispanic priming images (distinct from those seen in the scanner), making pain intensity and unpleasantness ratings after each stimulation. If at any point the pain became intolerable, the participant could ask the experimenter to remove the device. The participant was instructed to think about how each image related to their Hispanic (or US-American for English trials) culture when viewing the priming images. Finally, the participant completed one practice run of the semantic judgement task before being taken to the MRI scanner.

While being positioned in the scanner, during scanning, and when being removed from the scanner, all participants spoke directly with a single early bilingual MRI technician (with native fluency in both English and Spanish) who operated the scanner and maintained communication in the target language according to a script written by the experimenter. During scanning, participants were connected to physiology equipment (BIOPAC Systems Inc.) which recorded their electrodermal (EDA) and cardiac activity (Photoplethysmography; PPG) from sensors connected to the participant’s left foot. The thermode was connected via a Velcro strap to their left volar forearm. After being positioned in the scanner, the participant completed the Spanish run of the semantic judgement task (Figure S1). After a seven second resting baseline, instructions explaining the task were presented. The participant then completed 16 sentence trials in which they indicated whether each sentence was semantically plausible (i.e., “correct”, “
“*correcto”*) or not (“incorrect”, “*incorrecto”*). The side of the screen on which the “correct” and “incorrect” options appeared varied randomly across sentences, which were presented in random order. Additional seven second rest fixations occurred both at the middle and end of the run.

The participant then completed two Spanish runs of the pain rating task (Figure S2) with the thermode attached to a random one of four skin sites on their left forearm. The run began with a 10 second baseline fixation and a single “washout” heat stimulation (same parameters as described in Stimuli and Survey Measures) at a medium stimulus intensity (47°C) to habituate the skin site to heat stimulation. No ratings were collected for the washout stimulation. The participant then saw Spanish instructions explaining that they would receive a series of painful (but tolerable) thermal stimulations, rating each for the intensity and unpleasantness of the pain. A second instruction page explained that they would view images throughout the task and that they should describe in their head how the image related to their Hispanic culture. These written instructions, and short sentences reminding them to “remain still during the heat stimulation”, served to further engage the language context throughout the task. The participant received nine total non-washout stimulations making intensity and unpleasantness ratings after each, with the order of these ratings randomized for each trial. Nine Hispanic cultural priming images (Gianola et al., 2020) were interleaved between stimulations in a pseudorandomized fashion such that no more than two stimulations or two images occurred back-to-back. The priming images shown between stimulations served to 1) keep in mind the cultural context associated with the target language and 2) space out stimulations to prevent excessive heat exposure. Three more 10 second baseline periods occurred between stimulation and image viewing trials and to conclude the run. Between runs, the experimenter and MRI technician entered the scanner room to reposition the thermode to the next skin site. While doing so, the technician asked the participant to verbally rate their average pain throughout the run (0-10 scale). The second run of this pain rating task was also completed in Spanish.

After one run of sematic judgement and two runs of the pain rating task (i.e., halfway through the scan session), communication from the MRI technician and written instructions shifted to English. At that point the anatomical scan occurred (see *fMRI Data Acquisition and Preprocessing),* providing time for the participant to begin thinking in English. After the structural scan, the participant completed one run of the semantic judgement task and two runs of the pain rating task. All procedures were the same as described above, with the exception that all written instructions and communications were now in English, and the participant was told to think about how the cultural primes related to their US-American culture. After the final run of the pain rating task, the participant was removed from the scanner and taken back to the experiment room where they completed the same series of surveys in English as they had filled out in Spanish before scanning. Finally, they filled out a brief completion survey before being compensated for their time. The procedure for participants counterbalanced to the English-first condition underwent the same procedures as described above with the only difference being the reversal of the language condition and cultural priming (US-American vs Hispanic) images.

[INSERT FIGURE S1]

[INSERT FIGURE S2]

*fMRI Data Acquisition and Preprocessing:*

MRI data were collected on a Siemens Magnetom Vida 3.0T MRI (Erlangen, Germany) scanner using a 20 channel Siemens BM Head/Neck coil at the Neuroimaging Facility of the University of Miami (Miami, USA). Sixty axial slices covering the whole brain volume were acquired with a multiband factor of six and acceleration factor of two. A total of 212 volumes were collected per functional run of the semantic judgement task and 690 volumes per functional run of the pain rating task (TR = 600ms, TE = 30ms, flip angle = 50°, FOV = 248mm, slice thickness = 3.0mm, 3.0 x 3.0 x 3.0mm isometric voxels). Tasks did not begin and volumes for each functional run were not saved until steady state had been reached by the scanner. A T1-weighted anatomical image was acquired from each participant using a Magnetization Prepared Rapid Gradient Echo (MPRAGE) sequence (TI = 930ms, sagittal orientation, flip angle = 9°, field of view = 256mm, slice thickness = 1mm, 1.0 x 1.0 x 1.0mm isometric voxels).

Data preprocessing and statistical analyses were conducted using FSL version 5.0.9. The anatomical images were preprocessed with the following steps: cropping, reorientation to standard MNI orientation, registration to standard space using FLIRT (Greve & Fischl, 2009; Jenkinson et al., 2002; Jenkinson & Smith, 2001), and brain-extraction. Preprocessing steps applied to functional data included brain extraction, motion correction using MCFLIRT (Jenkinson et al., 2002), smoothing using a full-width at half maximum 6mm Gaussian kernel, removal of low frequency drift using a 90s high-pass filter estimated from the data in FSL (Smith et al., 2004), and convolution with a double-gamma hemodynamic response function. Functional images were co-registered to structural images and transformed into standard MNI space (images presented in radiologic orientation, with left brain on the right side). Temporal autocorrelation was estimated and corrected via prewhitening using FMRIB’s Improved Linear Model (Woolrich et al., 2001). FSL’s MCFLIRT (Jenkinson et al., 2002) was used for robust motion correction, which corrects for excessive head motion using rigid-body transformation across six standard motion parameters (translations and rotations along X, Y, and Z axes). Additionally, a confound matrix for each functional run was specified to regress out time points corrupted by significant motion. The DVARS metric (Power et al., 2012), which calculates the root mean square intensity difference between volume N and volume N+1, was used to define time points for motion regression.

Based on high mean displacement and numerous motions spikes larger than the voxel size across their functional runs, two participants (one female) were removed from all scanning and behavioral analyses. One of these subjects only completed half the scan due to discomfort in the scanning environment. Furthermore, partial fMRI task data was collected for three functional runs across two participants. Two heat pain trials during a Spanish run were lost for one male participant due to a thermode error leading to early termination of the scan. One female participant found the pain intolerable and chose to end two functional runs early, leading to a loss of one Spanish heat pain trial and five English heat pain trials. Full fMRI data were collected across all remaining functional runs and participants.

*ROI Construction and Calculation of Average Activity and NPS Response:*

A similar GLM analysis to that described in the main text for analyzing pain task brain activity was used to create the “semantic” region of interest (ROI) to test the “language as meddler” hypothesis. First-level analysis of the semantic judgement task contrasted the 4.5 second sentence reading and response period against baseline (including control regressors for other task components). Functional runs were combined at the second level with run language as a covariate. Task-evoked neural activity was averaged across participants with counterbalance order and cultural orientation as participant-level covariates. A map of regions showing greater sentence-evoked activity in Spanish (vs English) at FDR *q*<0.01 was multiplied with the Neurosynth.org association test map for “language” studies at FDR *q*<0.01 (Yarkoni et al., 2011). The latter map represented an automated meta-analysis of 1101 studies identifying regions preferentially activated by language tasks (compared to other conditions). The combined Neurosynth “language” and Spanish>English semantic judgement contrast maps were multiplied and binarized to produce a language localizer “semantic” ROI mask. This mask comprised areas demonstrating sensitivity to the language condition during semantic judgement within this sample, limited to regions generally agreed to engage in language processing (Friederici, 2011; Xie & Myers, 2018), primarily including the left inferior and superior frontal gyri (IFG, SFG) and anterior and posterior portions of superior temporal gyrus and temporal pole (Figure 1 blue).

Two additional ROIs were defined based only on meta-analytic maps obtained from Neurosynth.org (Yarkoni et al., 2011). The search term “attention” generated a map which synthesized activations from 1831 studies at FDR *q*<.01 comprising bilateral superior parietal lobule (SPL), premotor cortex, and right paracingulate gyrus (Figure 1 red). The term “somatosensory” generated a map at FDR *q*<0.01 based on 674 studies confined primarily to bilateral SI and SII into the middle cingulate (MCC), subcortical insulae, cerebellum, and thalamus (Figure 1 green). Both ROIs comprise regions which, across studies, showed significantly greater activation for tasks related to the search term compared to unrelated tasks. These masks were binarized and used to test the “language as spotlight” and “language as inducer” hypotheses, respectively.

The three ROI masks, defined in standard space, were converted into each participant’s native space and applied to *Z*-statistic images of individual heat trials, resulting in average BOLD activity within each ROI for each heat trial across participants. Additionally, variance inflation factors (VIF) for heat trials were calculated based on each trial’s design matrix, identifying trials likely affected by movement artifacts. ROI analyses were limited to those trials with VIF< 3.5 (96.85% of sample), a cutoff used in prior experimental pain research (Losin et al., 2020). Trial-level average BOLD activity for each ROI was treated as an outcome measure in mixed effects regression models parallel to the explicit pain rating models (see *Behavioral Data Analysis*). These analyses investigated if semantic-, attention-, and/or somatosensory-related regions were significantly activated during painful heat and whether such activity differed across language conditions and/or cultural orientations.

Beyond GLM analyses, the multivariate Neurologic Pain Signature (NPS) was applied to each participant’s single-trial pain-evoked BOLD responses. Each heat trial’s *Z*-statistic image was first transformed into standard space. Using the Canlab Core toolbox (<https://github.com/canlab/CanlabCore>) in Matlab (version R2020b), the dot product of NPS weights and each *Z*-statistic image produced one NPS score per heat trial. The same VIF calculation and exclusion process applied for ROI analyses was utilized for NPS data. Mixed effects models with NPS scores as the outcome fit to trials with VIF< 2.5 and VIF< 3.5 did not differ appreciably. Thus, models retaining a larger proportion of the data (n=1352 trials) are presented in the **Results**. These mixed effects models maintained the same structure as those described for the explicit pain ratings (see *Behavioral Data Analysis*) with fixed effects for control variables, language condition, cultural orientation, and their interaction, and random intercepts across participants.

**Supplementary Results:**

Several control variables were included as predictors of no interest in all pain outcome models to account for known influences of these factors on pain responses: functional run, stimulation skin site, counterbalance order, age, gender, trial number, and stimulation temperature. These variables were controlled for across all linear regression models presented in the main text.

Participant’s pain intensity and unpleasantness ratings after each heat stimulation provided points of comparison of sensory-discriminative and affective-motivational responses to pain, respectively. Both outcomes were significantly predicted by stimulation temperature. Across participants, a 1°C increase in stimulation temperature was associated with a ~1.8 (out of 10) point increase in reported pain intensity and unpleasantness. Furthermore, stimulations delivered to skin site 3 were rated 0.28 points less intense than those delivered to site 1, and stimulations received during the first pain task run in the second language (i.e. after a break from heat stimulations during the structural scan and second semantic judgement task run) were rated 0.22 points less unpleasant than those received during the first pain task run in the first language. Tables S1 and S2 display the statistical outcomes for fixed effects for all control variables, and random effects components in these models, respectively. Given the lack of significant interaction effects in the explicit pain outcome models, similar models were fit with the interaction term excluded (Table S3). The pattern of observed control effects did not differ appreciably across the two sets of models.

In a parallel set of mixed effects models, a similar series of control variables were found to predict the primary neural outcomes of interest. Specifically, pain-evoked BOLD activity in the semantic, somatosensory, and attention ROIs and pain-evoked NPS responses all increased significantly with stimulation temperature. NPS, somatosensory, and semantic ROI signal were reduced during stimulations delivered to skin site 3 (relative to site 1), whereas attention and somatosensory ROI responses were higher during stimulations delivered to site 4. Additionally, semantic and somatosensory ROI activity during pain were higher for females compared to males on average, in line with previously observed patterns of neural language processing between sexes (Burman et al., 2008)and see(Zaidi, 2010)for a review). NPS and somatosensory responses tended to be lower among older participants. Except for semantic ROI responses, all neural outcomes showed significant variation across functional runs. As these neural outcomes are calculated in arbitrary units, the parameter estimates for these models presented in Table S1 are standardized so they can be interpreted as the increase in the outcome (in standard deviation units) for one standard deviation increase in the predictor.

Overall, the reasonable pattern of control effects on the various pain outcomes, particularly strong and consistent temperature effects, demonstrate that the experimental pain manipulation worked as expected. The inclusion of these predictors in the tested models bolsters claims that any observed significant associations between pain outcomes and language condition or cultural metrics are not due to experimental confounds.

As an additional manipulation check, values of semantic ROI activity and NPS responses calculated during heat stimulations were compared to those obtained during other portions of the task. While semantic ROI activity averaged over the nine trials in each run did not vary significantly between the two rating types (*t*(155)= 0.71, *d*=0.06, *p*= .481; paired *t*-tests), it was significantly higher during both intensity (*t(*155)= –4.48, *d*= –0.36, *p*< .001) and unpleasantness (*t(*155)= –4.12, *d*= –0.33, *p*< .001) ratings compared to average pain-evoked ROI activity (during heat; Figure S3a). This result indicates that the semantic ROI’s activity reflects the additional language processing required to read and respond to the rating questions. Despite overall lower activity, semantic ROI activity during the heat was still significantly greater than zero (*t*(155)= 6.69, *p*< .001, single sample *t*-test), implying some relevant language processing (e.g., sub-vocal thinking about the pain) likely occurred during stimulations. As anticipated, NPS responses were significantly lower (and generally below zero) during both image viewing and unpleasantness rating (comparison conditions) compared to during heat stimulations, and this NPS response increased linearly with stimulation temperature (r= 0.30, p<.001; Figure S3b). Further, NPS responses during heat trials correlated significantly with participants’ reported intensity and unpleasantness ratings (*r*= 0.35 and *r*= 0.30, respectively, both p<.001). Overall, these initial tests indicated that NPS responses and semantic ROI BOLD signal were appropriately gauging relevant nociceptive and language processing neural activity, respectively.

[INSERT TABLE S1]

[INSERT TABLE S2]

[INSERT TABLE S3]

[INSERT FIGURE S3]

**References:**

Barratt, W. (2006). *The Barratt simplified measure of social status (BSMSS): Measuring SES*. <http://socialclassoncampus.blogspot.com/2012/06/barratt-simplified-measure-of-social.html>

Benet‐Martínez, V., & Haritatos, J. (2005). Bicultural identity integration (BII): components and psychosocial antecedents. *Journal of Personality, 73*(4), 1015-1050.

Burman, D. D., Bitan, T., & Booth, J. R. (2008). Sex differences in neural processing of language among children. *Neuropsychologia, 46*(5), 1349-1362.

Christodoulou, J. A., Del Tufo, S. N., Lymberis, J., Saxler, P. K., Ghosh, S. S., Triantafyllou, C., et al. (2014). Brain bases of reading fluency in typical reading and impaired fluency in dyslexia. *PLoS One, 9*(7), e100552.

Coghill, R. C., Sang, C. N., Maisog, J. M., & Iadarola, M. J. (1999). Pain intensity processing within the human brain: a bilateral, distributed mechanism. *Journal of neurophysiology, 82*(4), 1934.

Friederici, A. D. (2011). The brain basis of language processing: from structure to function. *Physiological reviews, 91*(4), 1357-1392.

Gianola, M., Llabre, M. M., & Losin, E. A. R. (2021). Effects of language context and cultural identity on the pain experience of Spanish–English bilinguals. *Affective Science, 2*(2), 112-127.

Gianola, M., Yepes, B. E., & Losin, E. A. R. (2020). Selection and Characterization of Cultural Priming Stimuli for the Activation of Spanish and English Cultural Mindsets among Hispanic/Latino Bilinguals in the United States. *Social Psychology*.

Greve, D. N., & Fischl, B. (2009). Accurate and robust brain image alignment using boundary-based registration. *NeuroImage, 48*(1), 1095-9572 (Electronic).

Jenkinson, M., Bannister, P., Brady, M., & Smith, S. (2002). Improved optimization for the robust and accurate linear registration and motion correction of brain images. *NeuroImage, 17*(2), 825-841.

Jenkinson, M., & Smith, S. (2001). A global optimisation method for robust affine registration of brain images. *Medical Image Analysis, 5*(2), 143-156.

John, O. P., & Srivastava, S. (1999). The Big Five trait taxonomy: History, measurement, and theoretical perspectives. *Handbook of personality: Theory and research, 2*(1999), 102-138.

Losin, E. A. R., Woo, C.-W., Medina, N. A., Andrews-Hanna, J. R., Eisenbarth, H., & Wager, T. D. (2020, 2020/02/03). Neural and sociocultural mediators of ethnic differences in pain. *Nature Human Behaviour*. <https://doi.org/10.1038/s41562-020-0819-8>

Luk, G., De Sa, E., & Bialystok, E. (2011). Is there a relation between onset age of bilingualism and enhancement of cognitive control? *Bilingualism: Language and Cognition, 14*(4), 588-595.

Marian, V., Blumenfeld, H. K., & Kaushanskaya, M. (2007). The Language Experience and Proficiency Questionnaire (LEAP-Q): Assessing language profiles in bilinguals and multilinguals.

Power, J. D., Barnes, K. A., Snyder, A. Z., Schlaggar, B. L., & Petersen, S. E. (2012). Spurious but systematic correlations in functional connectivity MRI networks arise from subject motion. *NeuroImage, 59*(3), 2142-2154.

Price, D. D., Mcgrath, P. A., Rafii, A., & Buckingham, B. (1983). The validation of visual analog scales as ratio scale measures for chronic and experimental pain. *PAIN, 17*(1), 45-56. <https://doi.org/10.1016/0304-3959(83)90126-4>

Riley 3rd, J. L., King, C. D., Wong, F., Fillingim, R. B., & Mauderli, A. P. (2010). Lack of endogenous modulation and reduced decay of prolonged heat pain in older adults. *PAIN, 150*(1), 153-160.

Smith, S. M., Jenkinson, M., Woolrich, M. W., Beckmann, C. F., Behrens, T. E. J., Johansen-Berg, H., et al. (2004). Advances in functional and structural MR image analysis and implementation as FSL. *NeuroImage, 23 Suppl 1*, S208-219. <https://doi.org/10.1016/j.neuroimage.2004.07.051>

Sorlie, P. D., Avilés-Santa, L. M., Wassertheil-Smoller, S., Kaplan, R. C., Daviglus, M. L., Giachello, A. L., et al. (2010). Design and implementation of the Hispanic community health study/study of Latinos. *Annals of epidemiology, 20*(8), 629-641.

Szapocznik, J., Kurtines, W. M., & Fernandez, T. (1980). Bicultural involvement and adjustment in Hispanic-American youths. *International Journal of Intercultural Relations, 4*(3-4), 353-365.

Woolrich, M., Brady, M., & Smith, S. M. (2001). Hierarchical fully Bayesian spatio-temporal analysis of FMRI data. *NeuroImage, 13*(6), 1053-8119.

Xie, X., & Myers, E. (2018). Left inferior frontal gyrus sensitivity to phonetic competition in receptive language processing: A comparison of clear and conversational speech. *Journal of Cognitive Neuroscience, 30*(3), 267-280.

Zaidi, Z. F. (2010). Gender differences in human brain: a review. *The open anatomy journal, 2*(1).

Zea, M. C., Asner-Self, K. K., Birman, D., & Buki, L. P. (2003). The Abbreviated Multidimentional Acculturation Scale: Empirical validation with two Latino/Latina samples. *Cultural Diversity and Ethnic Minority Psychology, 9*(2), 107.

**Figure Legend:**

Figure S1: Order and timing of events for the semantic judgement task. ISI-Interstimulus interval

Figure S2: Order and timing of pain rating task. Stimulations for a functional run were delivered to one of four skin sites on the left forearm (bottom corner) Note: cultural priming images are presented as if occurring separate from stimulation and rating periods, but images and stimulations were intermixed, with no more than two consecutive trials of a given type. ISI- Interstimulus interval

Figure S3: Manipulation check of neural response outcomes. a) Language responsive ROI activity during heat stimulations, intensity, and unpleasantness rating periods. b) NPS responses calculated from neural activity while viewing images, rating pain unpleasantness and receiving heat stimulations (divided across stimulation temperature). Red diamonds represent mean of each condition, while violin and box-and-whisker plots reflect distribution of responses. Y-axes on both graphs reflect arbitrary units with meaningful zero-points. N.S.- Not significant, ***p<.001
