# Supplementary material for: Does pain hurt more in Spanish? The neurobiology of pain among Spanish–English bilingual adults
Source: Soc Cogn Affect Neurosci. 2023 Dec 15;19(1):nsad074. doi: 10.1093/scan/nsad074 (PMC10868134; doi:10.1093/scan/nsad074)
Supplement: nsad074_Supp [file nsad074_supp.zip › scan-23-126-File012.docx]

**Table S1:** Parameter estimates and statistical tests for fixed effects of control variables across six primary pain rating task outcomes

| **Outcome** | | Slope | Standard Error | 95% Confidence Interval | *p*-value |
| --- | --- | --- | --- | --- | --- |
|  | Predictors |  |  |  |  |
| **Intensity Rating** | |  |  |  |  |
|  | Intercept* | 5.27 | 0.84 | 3.68 — 6.85 | <.0001 |
|  | Age (in years) | –0.04 | 0.03 | –0.10 — 0.02 | .255 |
|  | Counterbalance | –0.20 | 0.03 | –0.87 — 0.47 | .578 |
|  | Gender | –0.03 | 0.36 | –0.70 — 0.65 | .943 |
|  | Run 2 | 0.002 | 0.09 | –0.18 — 0.18 | .983 |
|  | Run 3 | –0.09 | 0.10 | –0.28 — 0.10 | .347 |
|  | Run 4 | –0.14 | 0.10 | –0.33 — 0.05 | .143 |
|  | Skin Site 2 | –0.03 | 0.09 | –0.22 — 0.15 | .741 |
|  | Skin Site 3* | –0.28 | 0.10 | –0.47 — –0.09 | .004 |
|  | Skin Site 4 | 0.11 | 0.10 | –0.08 — 0.29 | .264 |
|  | Temperature (°C)* | 1.79 | 0.04 | 1.72 — 1.87 | <.0001 |
|  | Trial | 0.01 | 0.01 | –0.02 — 0.03 | .479 |
| **Unpleasantness Rating** | |  |  |  |  |
|  | Intercept* | 4.32 | 0.98 | 2.48 — 6.16 | <.001 |
|  | Age (in years) | –0.03 | 0.04 | –0.10 — –0.04 | .444 |
|  | Counterbalance | 0.16 | 0.41 | –0.62 — 0.94 | .699 |
|  | Gender | 0.33 | 0.42 | –0.46 — 1.12 | .429 |
|  | Run 2 | 0.01 | 0.10 | –0.19 — 0.21 | .911 |
|  | Run 3* | –0.22 | 0.11 | –0.42 — –0.01 | .041 |
|  | Run 4 | –0.14 | 0.10 | –0.235 — 0.06 | .169 |
|  | Skin Site 2 | 0.14 | 0.10 | –0.06 — 0.34 | .166 |
|  | Skin Site 3 | –0.07 | 0.10 | –0.27 — 0.13 | .512 |
|  | Skin Site 4 | 0.19 | 0.10 | –0.01 — 0.39 | .064 |
|  | Temperature (°C)* | 1.82 | 0.04 | 1.74 — 1.91 | <.001 |
|  | Trial | 0.003 | 0.01 | –0.02 — 0.03 | .825 |
| **Attention ROI BOLD Signal** | |  |  |  |  |
|  | Age (in years) | –0.05 | 0.08 | –0.20 — 0.10 | .494 |
|  | Counterbalance | 0.03 | 0.08 | –0.12 — 0.17 | .731 |
|  | Gender | –0.15 | 0.08 | –0.29 — 0.00 | .062 |
|  | Run 2 | –0.04 | 0.03 | –0.10 — 0.02 | .160 |
|  | Run 3* | 0.10 | 0.03 | 0.04 — 0.15 | .002 |
|  | Run 4* | 0.09 | 0.03 | 0.03 — 0.15 | .003 |
|  | Skin Site 2 | 0.04 | 0.03 | –0.02 — 0.09 | .236 |
|  | Skin Site 3 | –0.04 | 0.03 | –0.10 — 0.02 | .232 |
|  | Skin Site 4* | 0.11 | 0.03 | 0.05 — 0.16 | <.001 |
|  | Temperature (°C)* | 0.07 | 0.03 | 0.03 — 0.12 | .002 |
|  | Trial | –0.03 | 0.02 | –0.08 — 0.02 | .199 |
| **Semantic ROI BOLD Signal** | |  |  |  |  |
|  | Age (in years) | –0.02 | 0.08 | –0.18 — 0.14 | .797 |
|  | Counterbalance | –0.03 | 0.08 | –0.19 — 0.13 | .737 |
|  | Gender* | –0.19 | 0.08 | –0.36 — –0.03 | .024 |
|  | Run 2 | –0.01 | 0.03 | –0.07 — 0.05 | .711 |
|  | Run 3 | –0.02 | 0.03 | –0.08 — 0.04 | .565 |
|  | Run 4 | 0.01 | 0.03 | –0.05 — 0.07 | .686 |
|  | Skin Site 2 | –0.01 | 0.03 | –0.07 — 0.04 | .630 |
|  | Skin Site 3* | –0.07 | 0.03 | –0.12 — –0.01 | .029 |
|  | Skin Site 4 | 0.02 | 0.03 | –0.04 — 0.08 | .446 |
|  | Temperature (°C)* | 0.13 | 0.02 | 0.08 — 0.18 | <.001 |
|  | Trial | –0.01 | 0.02 | –0.05 — 0.04 | .833 |
| **Somatosensory ROI BOLD Signal** | |  |  |  |  |
|  | Age (in years)* | –0.05 | 0.07 | –0.18 — 0.09 | .483 |
|  | Counterbalance | –0.02 | 0.07 | –0.15 — 0.12 | .819 |
|  | Gender* | –0.15 | 0.07 | –0.28 — –0.01 | .037 |
|  | Run 2 | –0.03 | 0.03 | –0.09 — 0.03 | .314 |
|  | Run 3 | 0.03 | 0.03 | –0.03 — 0.09 | .276 |
|  | Run 4* | 0.08 | 0.03 | 0.02 — 0.14 | .009 |
|  | Skin Site 2 | 0.02 | 0.03 | –0.04 — 0.08 | .473 |
|  | Skin Site 3* | –0.12 | 0.03 | –0.18 — –0.06 | <.001 |
|  | Skin Site 4* | 0.09 | 0.03 | 0.03 — 0.15 | .002 |
|  | Temperature (°C)* | 0.21 | 0.02 | 0.17 — 0.26 | <.001 |
|  | Trial | –0.01 | 0.02 | –0.06 — 0.03 | .619 |
| **NPS Response** | |  |  |  |  |
|  | Age (in years)* | –0.17 | 0.07 | –0.31 — –0.02 | .031 |
|  | Counterbalance | –0.06 | 0.07 | –0.20 — 0.09 | .434 |
|  | Gender | –0.11 | 0.07 | –0.25 — 0.04 | .153 |
|  | Run 2 | 0.02 | 0.03 | –0.04 — 0.07 | .584 |
|  | Run 3* | –0.06 | 0.03 | –0.12 — –0.004 | .037 |
|  | Run 4 | 0.01 | 0.03 | –0.05 — 0.06 | .755 |
|  | Skin Site 2 | –0.02 | 0.03 | –0.08 — 0.03 | .417 |
|  | Skin Site 3* | –0.15 | 0.03 | –0.21 — –0.10 | <.001 |
|  | Skin Site 4 | 0.01 | 0.03 | –0.04 — 0.07 | .693 |
|  | Temperature (°C)* | 0.31 | 0.02 | 0.26 — 0.35 | <.001 |
|  | Trial | –0.03 | 0.02 | –0.07 — 0.01 | .173 |

Note: Estimates for all ROI and NPS response outcomes have been standardized, while explicit pain rating models are unstandardized. Parameter estimates are rounded to two decimals and *p*-values are rounded to three decimals. Categorical variables for functional run and skin site are dummy coded. These parameter estimates can be interpreted as relative change compared to run 1 or skin site 1, respectively. Negative values for counterbalance represent relative increase in pain outcomes for the English-first condition. Negative values for gender represent higher pain outcomes in females. **p*<.05
