# Supplementary material for: Does pain hurt more in Spanish? The neurobiology of pain among Spanish–English bilingual adults
Source: Soc Cogn Affect Neurosci. 2023 Dec 15;19(1):nsad074. doi: 10.1093/scan/nsad074 (PMC10868134; doi:10.1093/scan/nsad074)
Supplement: nsad074_Supp [file nsad074_supp.zip › scan-23-126-File013.docx]

**Table S2:** Estimates of random effects parameters in the six pain outcome models

| **Outcome** | | Variance | Standard |
| --- | --- | --- | --- |
|  | Predictors |  | Deviation |
| **Intensity Rating** | |  |  |
|  | Participant (N=39) | 1.13 | 1.06 |
|  | Residual (N=1395) | 1.48 | 1.22 |
| **Unpleasantness Rating** | |  |  |
|  | Participant (N=39) | 1.55 | 1.24 |
|  | Residual (N=1395) | 1.73 | 1.32 |
| **Attention ROI BOLD Signal** | |  |  |
|  | Participant (N=39) | 450.50 | 21.22 |
|  | Residual (N=1352) | 1813.20 | 42.58 |
| **Semantic ROI BOLD Signal** | |  |  |
|  | Participant (N=39) | 727.70 | 26.98 |
|  | Residual (N=1353) | 2386.40 | 48.85 |
| **Somatosensory ROI BOLD Signal** | |  |  |
|  | Participant (N=39) | 310.10 | 17.61 |
|  | Residual (N=1352) | 1607.80 | 40.10 |
| **NPS Response** | |  |  |
|  | Participant (N=39) | 143.10 | 11.96 |
|  | Residual (N=1353) | 533.90 | 23.11 |

All values rounded to two decimals. All models showed significant χ^2^ statistics for random effects of participant (*p’*s<.0001)
