# Supplementary material for: Does pain hurt more in Spanish? The neurobiology of pain among Spanish–English bilingual adults
Source: Soc Cogn Affect Neurosci. 2023 Dec 15;19(1):nsad074. doi: 10.1093/scan/nsad074 (PMC10868134; doi:10.1093/scan/nsad074)
Supplement: nsad074_Supp [file nsad074_supp.zip › scan-23-126-File014.docx]

**Table S3:** Fixed effects parameter estimates and statistical tests for predictors of interest across behavioral pain outcome models excluding interaction term.

| **Outcome** | | Slope | Standard Error | 95% Confidence Interval | *p*-value |
| --- | --- | --- | --- | --- | --- |
|  | Predictors |  |  |  |  |
| **Intensity Ratings** | |  |  |  |  |
|  | Language* | 0.16 | 0.07 | 0.03 — 0.29 | .014 |
|  | Cultural Orientation | –0.41 | 0.30 | –0.97 — 0.16 | .168 |
| **Unpleasantness Ratings** | |  | 0.07 | –0.16 — 0.12 |  |
|  | Language | –0.19 |  |  | .786 |
|  | Cultural Orientation | –0.34 | 0.33 | –0.97 — 0.30 | .306 |

Positive language effects represent higher pain outcomes in Spanish. Positive cultural orientation effects reflect higher pain outcomes with increasing US-American orientation. **p*< .05
