# Supplementary figures and images for: Does pain hurt more in Spanish? The neurobiology of pain among Spanish–English bilingual adults
Source: Soc Cogn Affect Neurosci. 2023 Dec 15;19(1):nsad074. doi: 10.1093/scan/nsad074 (PMC10868134; doi:10.1093/scan/nsad074)

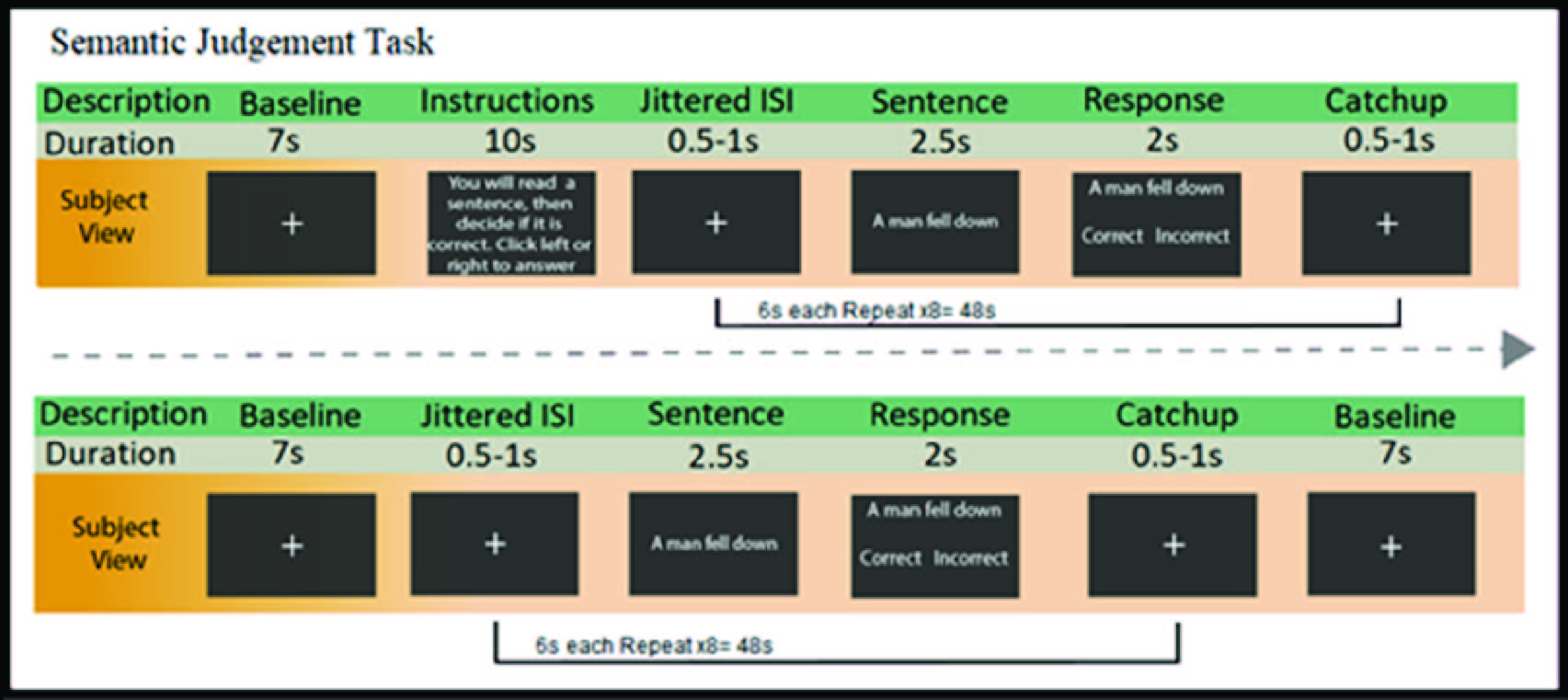

Supplement: nsad074_Supp [file nsad074_supp.zip › scan-23-126-File015.jpg]

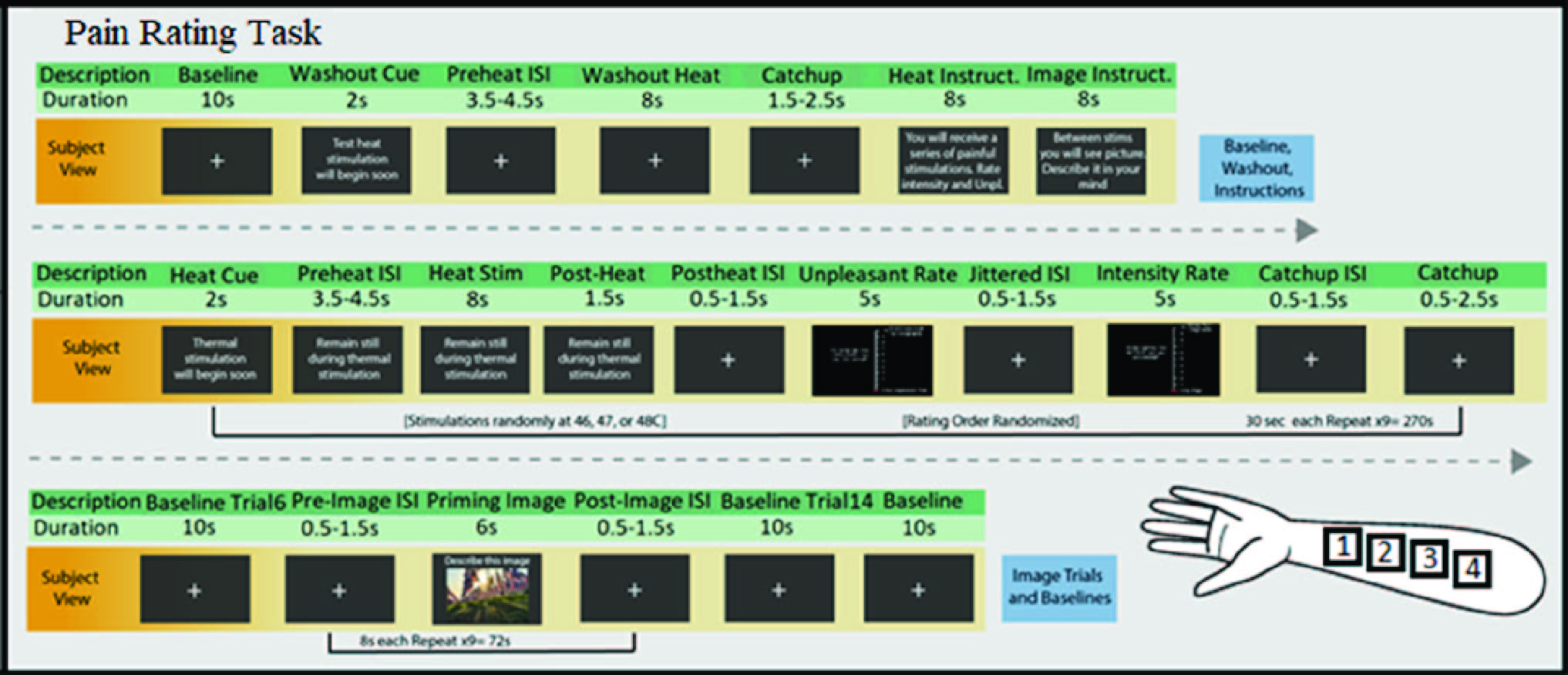

Supplement: nsad074_Supp [file nsad074_supp.zip › scan-23-126-File016.jpg]

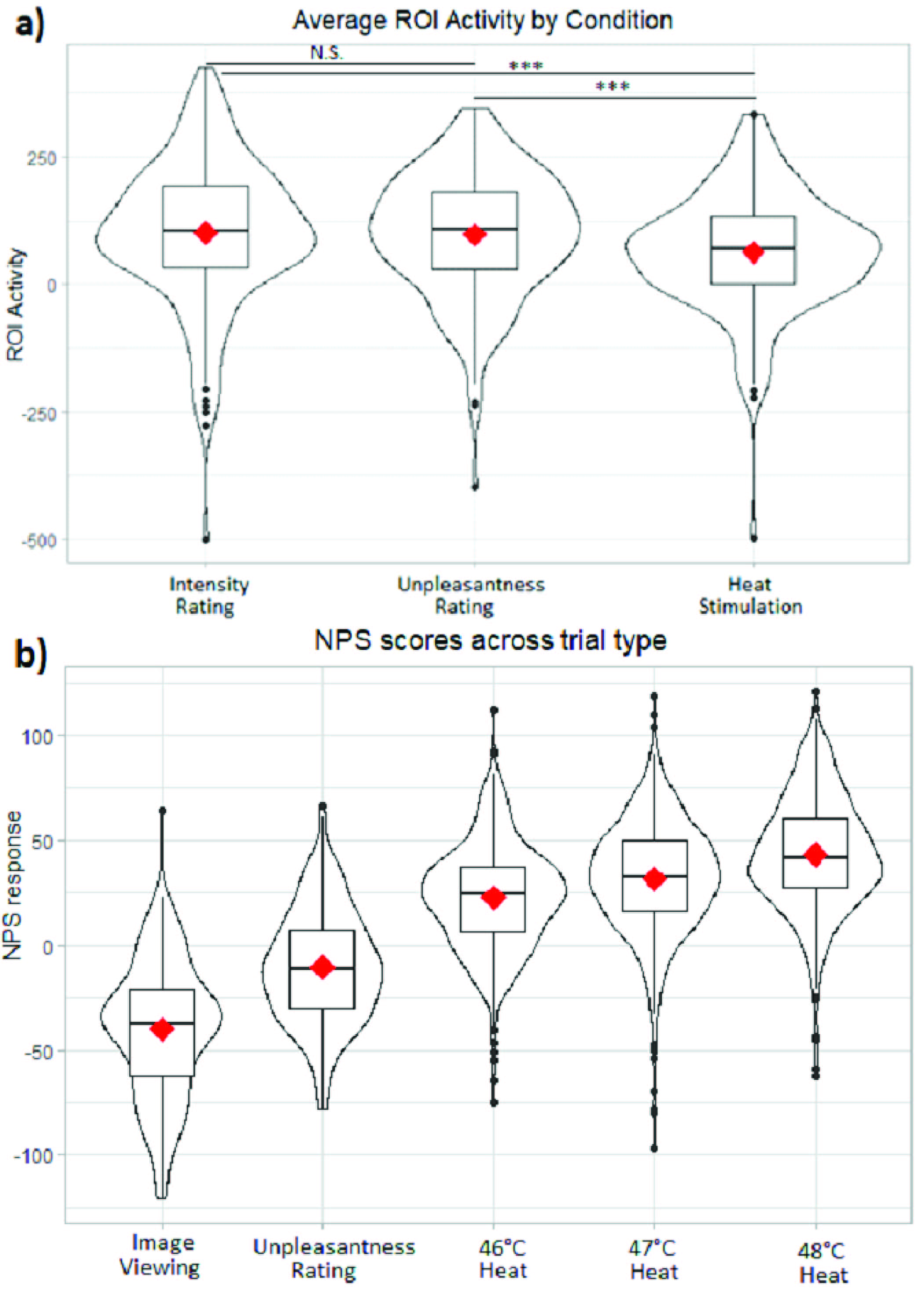

Supplement: nsad074_Supp [file nsad074_supp.zip › scan-23-126-File017.jpg]
